# One-stage posterior debridement with transverse process strut as bone graft in the surgical treatment of single-segment thoracic tuberculosis: A retrospective single-center study

**DOI:** 10.1097/MD.0000000000018022

**Published:** 2019-11-22

**Authors:** Xin-Jie Liang, Weiyang Zhong, Ke Tang, Zhengxue Quan, Xiao-Ji Luo, Dian-Ming Jiang

**Affiliations:** a Department of Pain Management; b Department of Orthopedic Surgery, The First Affiliated Hospital of Chongqing Medical University, Chongqing, China.

**Keywords:** bone graft, thoracic spine, transverse process, tuberculosis

## Abstract

This retrospective study investigated the effect of the novel bone graft transverse process strut (TPS) in single segmental thoracic spinal tuberculosis (TB) with the one-stage posterior approach of debridement, fusion, and internal instrumentation. Thirty patients treated in our department from March 2014 to October 2016 were retrospectively analyzed. Surgical time, blood loss, hospitalization time, drainage volume, and follow-up (FU) duration were recorded. The visual analog scale (VAS), Oswestry Disability Index (ODI), erythrocyte sedimentation rate (ESR), C-reactive protein (CRP), American Spinal Injury Association (ASIA) grade, segmental angle, and bone fusion were compared between preoperative and final FU. All the patients were followed for a mean 50.10 ± 25.10 months; the mean age, surgical time in minutes, blood loss, hospitalization time, and drainage volume were 46.23 ± 17.20 years, 195.08 ± 24.0 minutes, 280.77 ± 189.90 mL, 17.31 ± 4.23 days, 436.92 ± 193.81 mL, respectively. VAS and ODI scores were significantly improved at the final FU. The ESR and CRP returned to normal. All patients achieved bony fusion with a mean time of 5.85 ± 1.82 months and a mean segmental angle of 18.77 ± 2.49° preoperatively, which significantly decreased to 9.31 ± 1.54° at the final FU (*P* < .05). No complications, such as bone graft failure, pleural effusion, fistula, or wound infection were recorded except for cerebrospinal fluid leakage (one case), water electrolyte imbalance (5 cases), superficial infection (1 case), and mild intestinal obstruction (1 case). TPS as a bone graft is reliable, safe, and effective for segmental stability reconstruction for surgical management of single-segment thoracic spinal TB.

## 1 Introduction

Spinal tuberculosis (TB) is a common extrapulmonary form of an ancient disease that was first identified in Egyptian mummies.^[1–2]^ According to the World Health Organization, TB causes 1.81 million deaths in Asia annually, and China reports 78% new cases every year. Human immunodeficiency virus (HIV) infection, bacterial resistance, and population migration have caused the resurgence of all forms of TB. The spine is involved in 50% of osteoarticular TB cases; spinal involvement is especially dangerous, as it could result in vertebral body destruction, spinal deformity, and/or paraplegia. When the affected spine becomes unstable, the resulting deformity creates a risk of spinal cord compression.^[3–4]^ Thus, spinal TB should receive more attention. Traditionally, anti-TB drugs, other management strategies such as the use of a body brace or plaster bed, and a nutritious diet are surely effective. However, there are problems related to treatment because the principles of drug treatment vary according to our understanding of the treatment of pulmonary TB, the emergence of drug resistance and spinal TB in HIV-positive patients.^[3–4]^ Fortunately, surgery can be effectively used when spinal destruction results in spinal deformity and neurological deficits such as paraplegia.^[5–6]^

Surgical management is considered the most effective method of curing spinal TB. After debridement and decompression, many interbody bone grafts have been used to regain spinal stability, such as iliac crest or fibula graft and titanium mesh cages, which have their advantages and limitations.^[7–9]^ However, to date, no study reported TPS as a bone graft in the treatment of single-segment thoracic spinal TB. This study aimed to investigate the effect of TPS as a bone graft for restoring segmental stability.

## 2 Materials and methods

This study was approved by the Institutional Review Board of the First Affiliated Hospital of Chongqing Medical University and conducted in accordance with the Declaration of Helsinki. All participants provided written informed consent before their data were stored in our hospital database and used for study purposes.

From March 2014 to October 2016, in the spine unit of our department, a total of 30 patients with single-segment thoracic spinal TB were reviewed. The surgery procedure was performed by the same spine team.

### 2.1 Inclusion and exclusion criteria

Inclusion criteria: adult single-segment thoracic spinal TB, one-stage posterior approach, internal instrumentation and reconstruction, and severe pain and/or neurological deficits.

Exclusion criteria: active pulmonary TB, extrapulmonary TB, multiple-segment thoracic spinal TB, and cancer.

### 2.2 Preoperative management

Chemotherapy was administered as soon as the diagnosis was suggested clinically. Anti-TB drugs with the HREZ standard chemotherapy regimen that consists of isoniazid (5–10 mg/kg/day), rifampicin (10 mg/kg/day), ethambutol (15 mg/kg/day), and pyrazinamide (25 mg/kg/day) was administered 3 to 4 weeks before the operation.^[2,10]^ When the neurological deficits deteriorated progressively and failed the chemotherapy treatment, the time of drug treatment was shortened and the proper operative opportunity was considered. Surgical treatment was required when the ESR, CRP, and temperature recovered to normal or obviously decreased or the ESR decreased to below 60 mm/hour and CRP progressively decreased.

### 2.3 Surgical procedure

**Figure 1:**
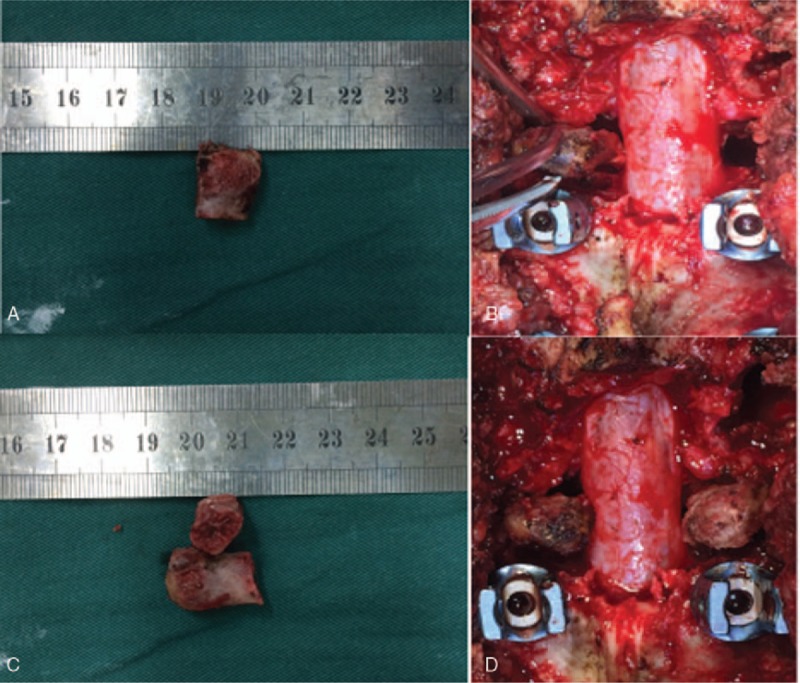
Photographs of one and two trimmed transverse processes before (A,C) and after (B,D) implantation.

After the administration of general anesthesia, the patients were placed in the prone position. The posterior spinal elements including the lamina, facet joints, and transverse processes were exposed through a midline incision. The pedicle screws were fixed based on imaging and C-arm X-ray findings, which were used to ensure its accuracy. Decompression and radical debridement were performed. The adjacent transverse process was cut off and the area was trimmed to a strut of round cortical bone and double-sided cancellous bone (Fig. 1). According to the space remaining after radical debridement, 1 or 2 or more transverse processes were implanted and appropriately forced with instrumentation locking. Streptomycin 1.0 g and isoniazid 0.2 g mixed with gelatin were used locally. Negative pressure drainage was managed postoperatively, and the specimens were sent for bacterial culture and pathology.

### 2.4 Postoperative care

Once the drainage volume was less than 50 mL/day, the drainage was removed. The patients continued the oral HREZ chemotherapy after surgery. One year later, the oral pyrazinamide was stopped. The patients continued 6-month regimens of HRE chemotherapy (12HREZ/12-18HRE). Rehabilitation therapist-guided ambulation exercise was started at 1 week postoperative. All patients were evaluated clinically and radiologically at 1 week, 3 months, 6 months, and 12 months after the operation and annually thereafter.

### 2.5 Follow-up index

For all patients, the data were observed perioperatively and during follow-up (FU):

(1) the operation time, surgical blood loss, hospitalization time, drainage, FU time and bone fusion time.
(2) Segmental angle. According to the Cobb method, the segmental angle was assessed by the angle of the fused vertebra.
(3) neurological function assessed by American Spinal Injury Association (ASIA) grade.
(4) VAS and ODI
(5) ESR and CRP.

The bone fusion was assessed using criteria of Bridwell et al with the X -ray and CT while necessary.

### 2.6 Statistical analysis

The statistical analysis was performed using Student *t* test with SPSS version 22.0 statistical software (SPSS, Inc, Chicago, IL). The results are expressed as mean ± SD. Differences with *P* values <.05 were considered statistically significant.

## 3 Results

### 3.1 Clinical assessments

The mean age, surgical time in minutes, blood loss, hospitalization time, drainage volume, and FU duration were 46.23 ± 17.20 years, 195.08 ± 24.0 minutes, 280.77 ± 189.90 mL, 17.31 ± 4.23 days, 436.92 ± 193.81 mL, and 50.10 ± 25.10 months, respectively (Table 1). The CRP, ESR, VAS, and ODI were significantly decreased at the final FU vs preoperatively (*P* < .05). The neurological deficits significantly improved at the final FU using ASIA grading (Table 2).

**Table 1:**
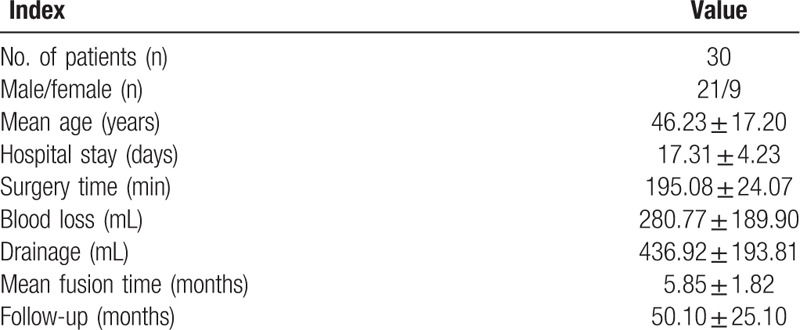
Patients’ general characteristics.

**Table 2:**
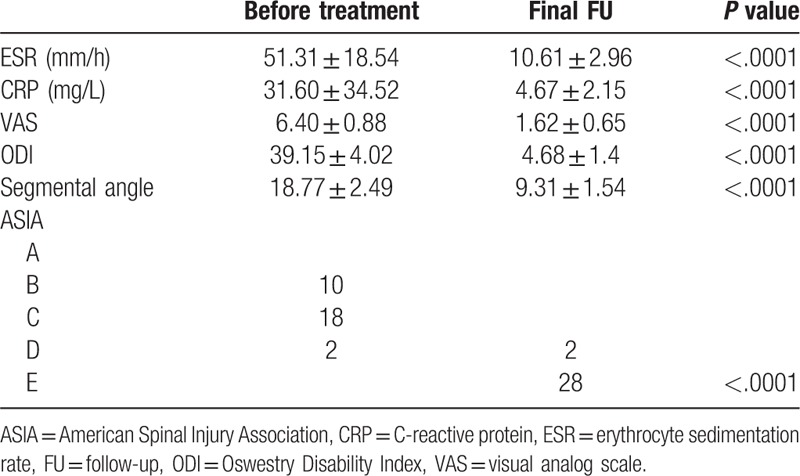
Comparison of preoperative and final FU related clinical and radiological outcomes.

### 3.2 Radiological assessments

**Figure 2:**
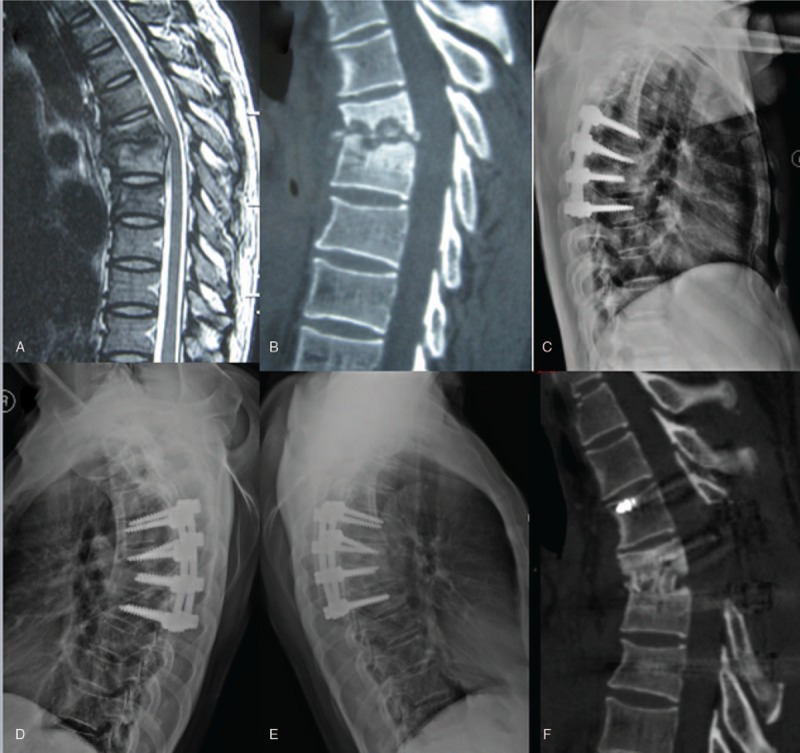
An 18-year-old man with thoracic spinal tuberculosis (T6-7) underwent posterior debridement and decompression combined with instrumentation. (A,B) Preoperative magnetic resonance imaging and CT showing bone destruction of the T6-7 vertebrae and compression of the spinal cord. (C,D) Seven-day and 6-month postoperative X-rays showing maintained correction. (EF) At 36 months’ follow-up, plain X-ray, and CT show solid bone fusion. CT = computed tomography.

**Figure 3:**
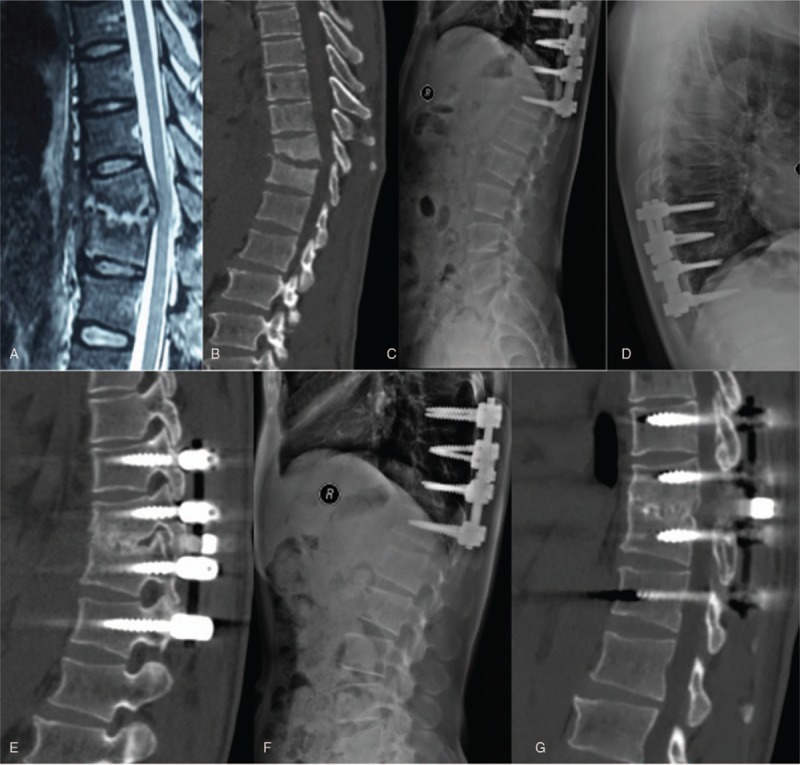
A 40-year-old man with thoracic spinal tuberculosis (T10-11) underwent posterior debridement and decompression combined with instrumentation. (A,B) Preoperative magnetic resonance imaging and CT revealing bone destruction of the T10-11 vertebrae and compression of the spinal cord. (C–E) At 7 days and 6 months postoperative, X-ray and CT demonstrate maintained correction. (F,G) At the 22-month follow-up, X-ray and CT show good bone fusion. CT = computed tomography.

The thoracic spinal TB was well cured and all patients achieved bony fusion at a mean time 5.85 ± 1.82 months. The mean segmental angle was 18.77 ± 2.49° preoperatively and significantly decreased to 9.31 ± 1.54° at the final FU (*P* < .05) (Figs. 2 and 3).

### 3.3 Complications

No complications related to the bone graft or internal instrumentation were observed postoperatively. Some postoperative complications occurred, such as cerebrospinal fluid leakage (1 case), water electrolyte imbalance (5 cases), superficial infection (1 case), and mild intestinal obstruction (1 case).

## 4 Discussion

The global incidence of TB is on the rise, especially in developing countries such as China and India. Spinal TB is the most common and severe form of osseous TB. Formal adequate and long-term anti-TB chemotherapy, bed rest, and supportive nutrition are the most essential therapies.^[10–11]^ Despite the efficacy of conservative regimens, surgery is indicated for patients affected by bone destruction, sequestration, a paraspinal or spine canal abscess, or nerve impairment. When aged patients have many comorbidities, it is challenging to overcome severe trauma such as significant blood loss, a longer operation time, and more severe complications associated with the anterior approach. A posterior pedicle screw system has been widely applied to correct deformities and stabilize unstable spinal segments, which effectively treats thoracic spinal disorders that lead to spinal instability and neurological impairments. Hence, therapeutic management of the TB entity has been modified to become more accurate and minimally invasive.^[12,13]^

After debridement and decompression, many interbody bone grafts are used to restore anterior and middle column stability. An iliac crest or fibula graft, considered the “gold standard,” can achieve a high bone healing rate. However, donor site complications such as persistent pain, hematoma, and unhealed wounds occur. Allografts can avoid complications but feature disadvantages associated with increased graft collapse rates and transmitted diseases. Titanium mesh cages filled with autograft have been extensively used and have high bone fusion rates. However, the problems of subsidence, stress shielding, and radio-opacity affect surgical plans.^[14–16]^ Thus, the current study aimed to identify new bone grafting method that can provide strut support and bone fusion to decrease the complication rate.

The transverse processes are the most prominent portions of the thoracic spine. Cui et al reported that the morphologies of the thoracic and lumbar vertebrae differed significantly. Except for the L3 to L5 segment, compared to the lumbar vertebra, the thoracic transverse processes are longer, thicker, higher, and larger. He concluded that the thoracic transverse process could be used as bone grafts.^[17–18]^ Kunkel et al demonstrated that disc thickness varied from 4.5 to 7.2 mm, with the middle disc height being approximately 22.7% greater and the mean vertebral body height varied from 14.88 to 21.96 mm.^[19]^ Panjabi and Tan also detailed the quantitative 3-dimensional anatomy of thoracic vertebrae.^[20–21]^ Anatomically, the transverse process satisfies the need for a bone graft if the bone damage incurred by the infected vertebral body did not exceed 1/2 of the vertebral height.

Use of the TPS as the bone graft has the following advantages. First, compared with the iliac crest or fibula graft in previous studies,^[22–28]^ the TPS could reduce trauma and bleeding, shorten surgical and hospitalization times, decrease postoperative drainage volumes, and postoperative complication rates. The VAS and ODI were also improved during FU; thus, the patients recovered soon after surgery. The transverse processes were in the surgical exposure area during use of the posterior approach, which retained the integrity and original attachment structure of the ribs and reduced bleeding and trauma. Second, in our study, all patients achieved bony fusion at a mean 5.85 ± 1.82 months. The mean segmental angle decreased significantly from 18.77 ± 2.49° preoperative to 9.31 ± 1.54° at the final FU. The TPS could provide good support, strength, and fusion properties that effectively restored the spinal stability. Additionally, the transverse process, as an autogenous bone, has a 3-sided cortical bone structure and was properly trimmed into a double-side cancellous bone graft. The use of 1 to 3 TPS was suitable for the bone defect space, which both reduces implant difficulty and ensures a large contact area between the bone graft and the end plate, ensuring good bony fusion.

We believe that use of the TPS for the surgical indications of single-segment thoracic spinal TB debridement satisfies the need for a graft if the bone damage induced by the infected vertebral body does not exceed 1/2 of the vertebral height.

## 5 Conclusion

Our study results show that use of the TPS achieved segmental stability in the surgical management of single-segment thoracic spinal TB, resulting in satisfactory bone fusion, good kyphosis deformity correction, and spinal stability restoration, is a reliable, safe, and effective bone grafting method. However, since this was a small-sample retrospective study, prospective randomized studies with long-term FU periods are needed to validate our findings.

## Author contributions

**Conceptualization:** Xinjie Liang, Weiyang Zhong.

**Data curation:** Xinjie Liang, Ke Tang.

**Formal analysis:** Ke Tang, Zhengxue Quan.

**Investigation:** Zhengxue Quan, Xiaoji Luo, Dianming Jiang.

**Methodology:** Ke Tang, Xiaoji Luo, Dianming Jiang.

**Resources:** Weiyang Zhong.

**Writing – original draft:** Xinjie Liang, Weiyang Zhong.

**Writing – review & editing:** Xinjie Liang, Weiyang Zhong.
